# A Novel Prognostic Marker for Primary CNS Lymphoma: Lactate Dehydrogenase-to-Lymphocyte Ratio Improves Stratification of Patients Within the Low and Intermediate MSKCC Risk Groups

**DOI:** 10.3389/fonc.2021.696147

**Published:** 2021-08-03

**Authors:** Yuting Gao, Li Wei, Seok Jin Kim, Liang Wang, Yingzhi He, Yanfang Zheng, Luca Bertero, Alessia Pellerino, Paola Cassoni, Luca Tamagnone, Prochazka Katharina Theresa, Alexander Deutsch, Huien Zhan, Jing Lai, Yao Wang, Hua You

**Affiliations:** ^1^Department of Oncology, Affiliated Cancer Hospital & Institute of Guangzhou Medical University, Guangzhou, China; ^2^NHC Key Laboratory of Birth Defects and Reproductive Health, Chongqing Population and Family Planning Science and Technology Research Institute, Chongqing, China; ^3^Division of Hematology-Oncology, Department of Medicine, Samsung Medical Center, Sungkyunkwan University School of Medicine, Seoul, South Korea; ^4^Department of Hematology, Beijing Tongren Hospital, Capital Medical University, Beijing, China; ^5^Department of Hematology, ZhuJiang Hospital of Southern Medical University, Guangzhou, China; ^6^Department of Oncology, ZhuJiang Hospital of Southern Medical University, Guangzhou, China; ^7^Pathology Unit, Department of Medical Sciences, University of Turin, Torino, Italy; ^8^Department of Neuro-Oncology, University and City of Health and Science Hospital, Torino, Italy; ^9^Università Cattolica del Sacro Cuore, Department of Life Sciences and Public Health, Rome, Italy; ^10^Fondazione Policlinico Universitario “A. Gemelli”- IRCCS, Rome, Italy; ^11^Clinical Department of Hematology, Medical University of Graz, Graz, Austria; ^12^Department of Hematology, The First Affiliated Hospital of Jinan University, Guangzhou, China

**Keywords:** primary central nervous system lymphoma, lactate dehydrogenase-to-lymphocyte ratio, prognostic parameter, Memorial Sloan Kettering Cancer Center (MSKCC) score, neutrophil-to-lymphocyte ratio

## Abstract

**Background:**

Primary central nervous system lymphoma (PCNSL) is a highly aggressive and rare extranodal non-Hodgkin lymphoma (NHL). The MSKCC and the IELSG scores represent the most widely used prognostic models, but many changes have occurred in therapeutic protocols since their development. Moreover, many PCNSL patients cannot be classified using the IELSG score. We thus aimed to create a novel, effective and feasible prognostic model for PCNSL.

**Methods:**

We included 248 PCNSL patients diagnosed with PCNSL. Our primary endpoint was the overall survival (OS) and we used the receiver operating characteristic (ROC) analysis to determine the optimal prognostic cut-off value for LLR (lactate dehydrogenase-to-lymphocyte ratio), neutrophil-to-lymphocyte ratio (NLR) and derived neutrophil-to-lymphocyte ratio (dNLR). Variable associated with OS were evaluated by univariate and multivariate analyses. 124 out of 248 patients were randomly selected as the internal validation cohort.

**Results:**

By univariate analysis, an age >60 years, Eastern Cooperative Oncology Group performance status (ECOG PS) >1, treatment with radiotherapy alone, high-risk groups of Memorial Sloan Kettering Cancer Center (MSKCC) score, NLR >4.74, dNLR >3.29, and LLR >166.8 were significantly associated with a worse OS. By multivariate analysis, the MSKCC score and LLR were confirmed as independent prognostic parameters for poorer OS. OS, however, was not significantly different between low- and intermediate-risk groups according to the MSKCC score, while LLR proved to be prognostically relevant and was thus used to develop a novel, effective three-tier PCNSL scoring system. Of 124 patients, 84 patients with survival data and LLR data were successfully validated by newly established PCNSL LLR scoring system.

**Conclusions:**

In the present study, we demonstrate that a high LLR represents an independent unfavorable prognostic parameter in PCNSL patients which can be integrated into an effective prognostic model.

## Introduction

Primary central nervous system lymphoma (PCNSL) is defined as a pathologically confirmed primary lymphoma of the central nervous system, arising within the brain, leptomeninges, spinal cord and eyes, without systemic involvement; it represents a highly aggressive and rare extranodal non-Hodgkin lymphoma (NHL) with a poor prognosis. Around 90%–95% PCNSLs are histologically classified as diffuse large B-cell lymphomas (DLBCL) (1). Although high-dose systemic methotrexate (HD-MTX)-based chemotherapy and whole brain radiotherapy (WBRT) have improved the outcomes of this disease in the last decade, its prognosis is still unsatisfactory (2). Present median overall survival is 36.9–46 months, with a five-year survival rate ranging from 22.3% to 32% (3–5).

Two scoring systems are commonly used to predict the outcome of PCNSL patients: the International Extranodal Lymphoma Study Group (IELSG) score (6) and the Memorial Sloan Kettering Cancer Center (MSKCC) score (7). The first one was validated in 378 PCNSL patients treated at 23 cancer centers from five countries between 1980 and 1999. However, only 105 patients had complete data and were included in the model and the median follow-up was 24 months only. In this study, the IELSG found that an age >60 years, Eastern Cooperative Oncology Group performance status (ECOG PS) >1, high level of lactate dehydrogenase (LDH), elevated cerebrospinal fluid protein concentration and involvement of deep regions of the brain were significantly associated with a worse outcome. The 2-year overall survival (OS) was 80%, 48% and 15% for patients with 0 to 1, 2 to 3, and 4 to 5 unfavorable parameters, respectively (6). The MSKCC score was developed within a study including 338 PCNSL patients recruited during 1983 to 2003. Age and Karnofsky performance score (KPS) were the only two variables included in the prognostic model, and they were used to stratify participants into low-, intermediate-, and high-risk groups (characterized by: age <50 irrespective of KPS, age ≥50 and KPS ≥70, age ≥50 and KPS <70), which correlated with median OS of 8.5, 3.2 and 1.1 years, respectively (7). Despite the clinical usefulness of the MSKCC and IELSG prognostic models, they should be further improved and updated considering the progress in PCNSL treatments (e.g. rituximab availability) (6). Moreover, a major limitation of the MSKCC prognostic model is an intrinsic selection bias for being a single institution study (7). Gene mutation status and/or gene expression profiling combined with or without the prognostic models (8) were also investigated to predict the clinical outcomes of PCNSL. However, the use of molecular markers is always associated with high costs, laboratory efforts, and time-consuming procedures, which are not routinely available in many clinical units and diagnostic laboratories (9). Therefore, there is a growing need for cost-effective and easily applicable prognostic markers that might help to improve the prognostic accuracy of existing models (9).

Recent data showed that inflammation actively participates in tumor progression, shaping the microenvironment, and promoting cancer cell proliferation, survival and migration (10). A series of parameters related to systemic inflammation have been identified as prognostic factors in lymphomas, including the lymphocyte count (11), levels of C-reactive protein, erythrocyte sedimentation rate (ESR), neutrophil-to-lymphocyte ratio (NLR) (12), derived neutrophil-to-lymphocyte ratio (dNLR) (13), and lymphocyte-to-monocyte ratio (LMR) (14). Peripheral blood count and biochemical markers, which are simple and widely available tests, may be a surrogate of the inflammatory and immune alterations caused by lymphomas. However, in a recently published study, analysis of inflammation markers alone has not proven capable of predicting the clinical therapeutic outcomes (15). Thus, tumor burden-associated markers, e.g. lactate dehydrogenase, and inflammation markers such as lymphocyte counts have been combined in the present study to develop a novel prognostic model.

In fact, lactate dehydrogenase-to-lymphocyte ratio (LLR) is an established prognostic parameter in extranodal natural killer/T cell lymphoma (ENKTL) (16), metastatic renal cell carcinoma (mRCC) (17) and DLBCL patients (18). Moreover, Dai et al. developed a novel prognostic model based on Ann Arbor stage, β2-microglobin to lymphocytes ratio (βLR) and LLR which resulted an independent prognostic parameter in early stage ENKTL patients (16). Tao et al. revealed that patients with a LLR ≥ 150 had shorter median progression free survival (PFS) and OS (PFS 9 months *vs* 18 months; OS 21 months *vs* 46 months) in mRCC patients treated with tyrosine kinase inhibitors (17). In DLBCL patients, a high LLR was associated with poor 5-year PFS and OS (PFS 45% *vs* 78%; OS 56% *vs* 86%) (18). However, the prognostic significance of LLR in PCNSL patients has never been explored, and thus we set out to evaluate the hypothesis that LLR could be exploited to create a novel, effective and feasible prognostic model for PCNSL.

## Methods

### Patients

We retrospectively collected data from 6 centers in 4 countries, the Affiliated Cancer Hospital & Institute of Guangzhou Medical University in China, ZhuJiang Hospital of Southern Medical University in China, the First Affiliated Hospital of Jinan University in China, Medical University of Graz in Austria, Samsung Medical Center in Korea, and University of Turin in Italy. We included 248 immunocompetent patients with PCNSL diagnosed between November 2004 and December 2019. All clinico-pathological parameters such as the histologically confirmed diagnosis of PCNSL (DLBCL histotype), gender, age, cell of origin categories (GCB and ABC subtype according to the Hans algorithm), survival data, and laboratory results were retrieved from medical records in their respective hospitals and reviewed. All the patients included in this study fulfilled the following criteria: (1) disease localized exclusively in the brain, leptomeninges, spinal cord and eyes without systemic involvement; (2) seronegative for human immunodeficiency virus; (3) none history of immunosuppression or organ transplantation; (4) none other malignancies diagnosed during the observation period; (5) none previous anti-cancer treatment; (6) adequate clinical, laboratory, and follow-up data available. All procedures involving human participants were performed in accordance with the ethical standards of the institutional and/or national research committees and according to the 1964 Helsinki declaration and its later amendments or comparable ethical standards. Considered the retrospective nature of the study and that patients’ data had been de-identified in the dataset, our institutional ethics review board approved the study and waived the need for informed consent.

### Pre-Treatment Systematic Inflammation-Based Prognostic Parameters

Pre-treatment laboratory parameters, including the neutrophil count, derived neutrophil count, lymphocyte count, and serum levels of LDH, were obtained before treatment initiation. These data were calculated the median and interquartile range. NLR was calculated as the absolute neutrophil count divided by the absolute lymphocyte count, dNLR was determined as neutrophil count divided by the result of leukocyte count minus neutrophil count, LLR was determined as the serum level of LDH divided by the absolute lymphocyte count.

### Internal Validation

124 out of 248 patients with PCNSL were randomly selected as the internal validation cohort. The established PCNSL model was examined in the validation cohort.

### Clinical Outcomes and Statistical Analysis

We evaluated the treatment responses as CR (complete response), PR (partial response), SD (stable disease), and PD (progressive disease) according to The International Working Group Recommendations for Response Criteria of non-Hodgkin’s lymphoma (19). Dates of death were obtained from clinical records, official civil registries or by telephone calls to patients’ relatives. OS was calculated in months and defined as the time from the date of first diagnosis to either the date of death from any cause or the last follow-up date. Receiver-operating-characteristics (ROC) analysis was adopted to define the optimal cut-off value for LLR, NLR, and dNLR. Survival curves were analyzed by the log rank test and Kaplan–Meier method. The prognostic capability (in terms of OS) of the included variables was evaluated by univariate Cox analysis. Significant variables (*P-value* < 0.05) were included into the multivariate analysis using the forward conditional Cox regression model. All the statistical analyses were performed using the SPSS 16.0 software (IBM, Armonk, New York, US) and *P* values (two-tailed) < 0.05 were considered to be statistically significant.

## Results

### Patient Characteristics

**Figure 1:**
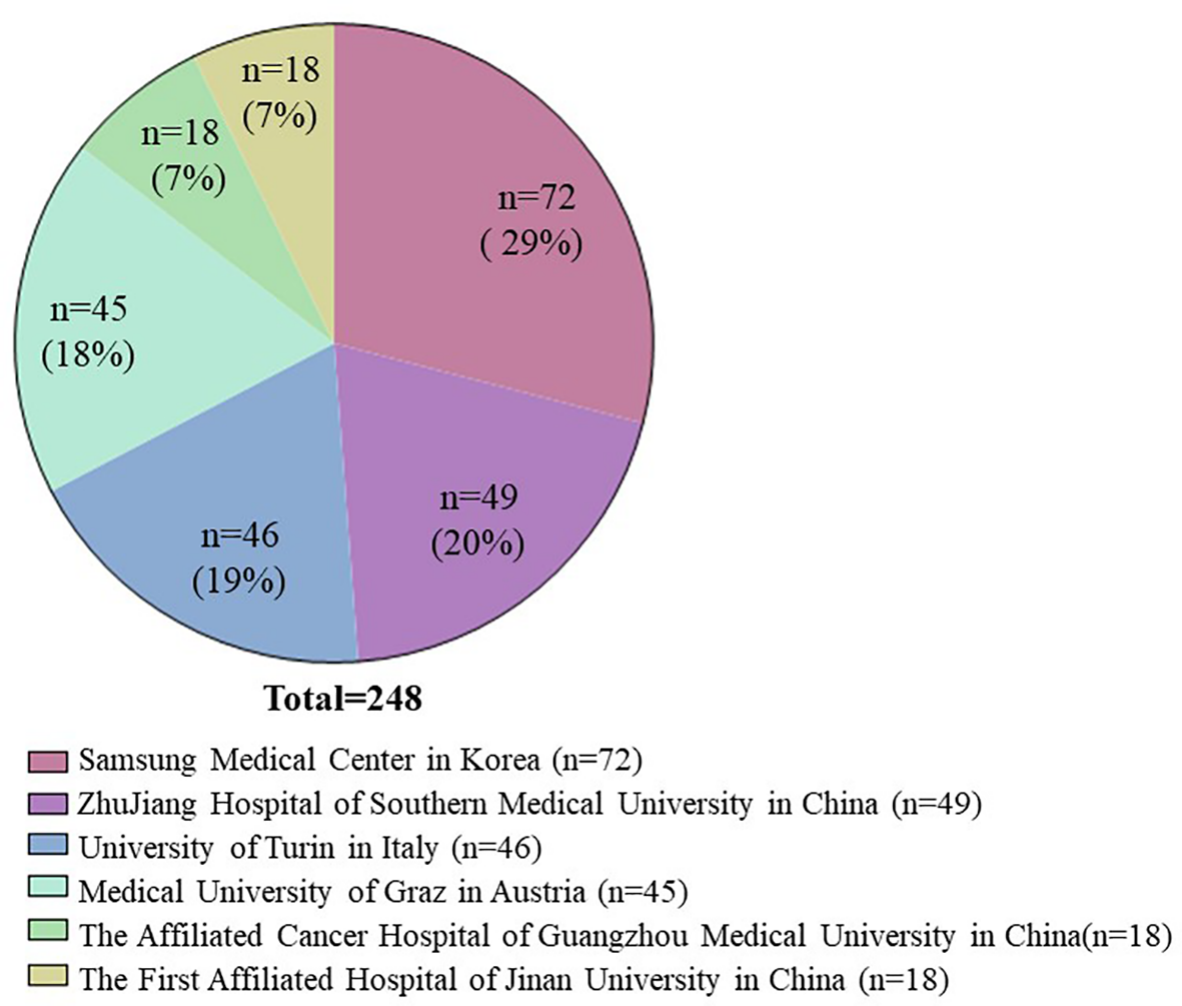
The percentage of patients participating in each institution.

We included 248 patients with confirmed PCNSL, diagnosed in 6 cancer centers. The percentage of patients participating in each institution is shown in **Figure 1**. Patients’ median age at the time of diagnosis was 59 years (range: 21–86), and 56.0% were males. An ECOG PS of 0–1 was seen in 52.8% of the subjects (131/248). According to the MSKCC scoring system, most patients (58.9%, 146/248) were classified as intermediate risk, 54 patients as low risk (21.8%) and 48 patients as high risk (19.3%). Data regarding the cell of origin were available for 153 patients, 117 of which had non germinal center B-cell subtype lymphomas. Systemic B symptoms were observed in a small fraction of the patients (6/131), while elevated LDH levels were observed in 117 cases (47.2%). Of all 248 patients, 196 patients had EBV status data, but only 3 patients were EBV tissue-positive cases. Baseline characteristics are shown in **Table 1**.

**Table 1:** Clinico-pathological characteristics of 248 patients with PCNSL.

Parameter	N(%)
Age	59 (21-86)
≤60	127 (51.2)
>60	121 (48.8)
Gender	
Male	139 (56.0)
Female	108 (43.5)
ECOG PS	
≤1	131 (52.8)
>1	116 (46.8)
Cell of origin	
GCB	36 (14.5)
Non-GCB	117 (47.2)
Epstein-Barr virus	
Positive	3 (1.5)
Negative	193 (98.5)
B symptoms	
Yes	6 (2.4)
No	125 (50.4)
LDH	
≤250 U/L	103 (41.5)
>250 U/L	117 (47.2)
Treatment	
Chemotherapy	169 (68.1)
Radiotherapy	9 (3.6)
CMT+RT	33 (13.3)
First-line chemotherapy	
HD-MTX	173 (85.6)
Others	29 (14.4)
MSKCC	
Low	54 (21.8)
Intermediate	146 (58.9)
High	48 (19.3)

ECOG PS, Eastern Cooperative Oncology Group performance status; LDH, lactate dehydrogenase; GCB, germinal center B cell; CMT, chemotherapy; RT, radiotherapy; HD-MTX, high-dose methotrexate; MSKCC, Memorial Sloan Kettering Cancer Center.

### Treatment Modalities and Response

Therapeutic data of 211 patients were available. Radiotherapy (RT) alone was performed in 9 patients (3.6%), a combination of RT and chemotherapy (CMT) was performed in 33 patients (13.3%), and CMT alone was performed in 169 patients (68.1%). Methotrexate (MTX) was the most commonly used drug (n = 173), followed by alkylating agents (n = 124, including procarbazine, thiotepa, cyclophosphamide and temozolomide), and cytarabine (n = 35). Chemotherapy regimens were generally divided into HD-MTX-based regimens (n = 173) and non-HD-MTX regimens (n = 29). Non-HD-MTX regimens included cyclophosphamide, doxorubicin, vincristine, and prednisone (CHOP) or CHOP-like regimens, prednisolone alone or alkylating agents-based regimens. In addition, 49 (23.2%) patients received rituximab and 5 (2.4%) patients received autologous hematopoietic stem cell transplantation during treatment. After the initial treatment, 92 (43.6%) achieved CR, 62 (28.9%) achieved PR, 7 (3.3%) experienced SD, 29 (13.7%) had PD and in 22 (10.4%) response data were unavailable.

### Determination of Cut-Off Values

**Figure 2:**
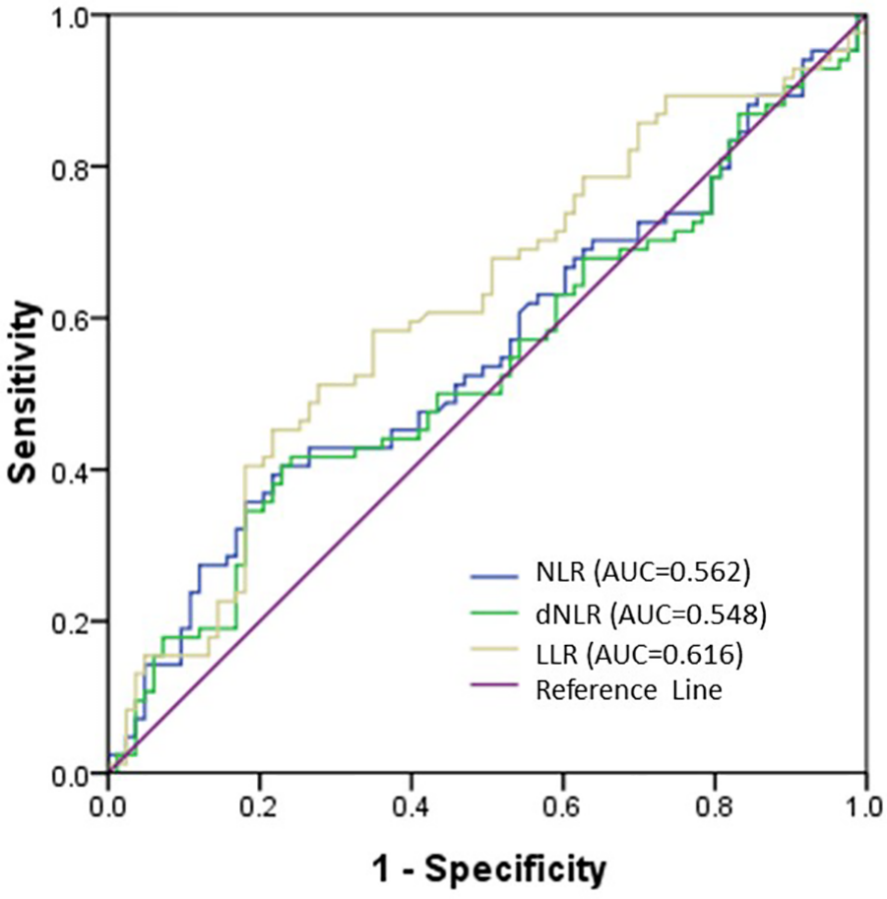
Receiver operating characteristic (ROC) curve of NLR, dNLR and LLR. The area under the ROC curve (AUC) of LLR was 0.616, which was preferable than the NLR (0.562) and dNLR (0.548) AUCs.

In this study, we used OS as the endpoint of interest and ROC analysis was performed to calculate the optimal cut-off value for LLR, NLR, and dNLR. The area under the ROC curve for LLR, NLR, and dNLR were 0.616, 0.562, and 0.548, respectively (**Figure 2**), and the optimal cut-off values corresponding to the maximum joint sensitivity and specificity were 166.8, 4.74, and 3.29, respectively (**Table 2**).

**Table 2:** Determination of the optimal cut-off values.

Marker	Cut-off values	AUC	95% CI	*P value*
NLR	4.74	0.562	0.478—0.646	0.15
dNLR	3.29	0.548	0.463—0.632	0.27
LLR	166.80	0.616	0.530—0.701	0.01

NLR, neutrophil-to-lymphocyte ratio; dNLR, derived neutrophil-to-lymphocyte ratio; LLR, lactate dehydrogenase-to-lymphocyte ratio; AUC, area under receiver operating curve; CI, confidence interval.

### Prognostic Parameters

Using univariate analysis and Cox regression model, we assessed the impact of LLR and of clinical characteristics on the OS. Univariate analysis identified several potential prognostic factors for poor OS: Age > 60 (hazard ratio [HR] 1.735, *P* = 0.007), ECOG PS > 1 (HR 1.888, *P* = 0.001), treatment with radiotherapy alone (HR 4.807, *P* = 0.010), high-risk group of MSKCC score (HR 2.687, *P* = 0.004), NLR > 4.74 (HR 1.634, *P* = 0.023), dNLR > 3.29 (HR 1.568, *P* = 0.039), and LLR > 166.8 (HR 1.710, *P* = 0.016) (**Table 3**). In multivariate analysis, both LLR > 166.8 and the MSKCC scores were independent prognostic parameters for OS (*P* = 0.015 and *P* = 0.004, respectively) (**Table 4**). Conversely, age, ECOG PS, treatment, NLR, and dNLR were not significant.

**Table 3:** Univariate analysis of variables associated with overall survival.

Parameter		(median, IQR)	HR	(95%CI)	*P value*
Age	≤60	(52, 8)			***0.007***
	>60	(68, 10)	1.735	(1.163,2.588)	
Gender	Male	–			0.705
	Female	–	0.927	(0.624,1.375)	
ECOG PS	≤1	–			***0.001***
	>1	–	1.888	(1.278,2.789)	
B symptom	Yes	–			0.480
	No	–	0.599	(0.145,2.483)	
Cell of origin	GCB	–			0.088
	Non GCB	–	0.555	(0.282,1.091)	
EBV	Negative	–			0.481
	Positive	–	2.047	(0.280,14.989)	
Treatment	Chemotherapy	–	–	–	***0.010***
	Radiotherapy	–	4.807	(1.724,13.407)	
	CMT+RT	–	1.250	(0.676,2.311)	
MSKCC	Low	–	–	–	***0.004***
	Intermediate	–	1.461	(0.838,2.545)	
	High	–	2.687	(1.446,4.991)	
LDH	≤250 U/L	(179, 53)			0.255
	>250 U/L	(384, 157)	1.289	(0.823,1.997)	
NLR	≤4.74	(2.19, 1.46)			***0.023***
	>4.74	(7.28, 5.56)	1.634	(1.069,2.498)	
dNLR	≤3.29	(1.66, 0.96)			***0.039***
	>3.29	(5.06, 3.27)	1.568	(1.023,2.405)	
LLR	≤166.8	(110.75, 50.09)			***0.016***
	>166.8	(242.05, 195.36)	1.710	(1.105,2.646)	
Neutrophil	≤2.0×10^9^/L	(1.76, 0.26)			0.644
	>2.0×10^9^/L	(5.05, 3.76)	1.593	(0.222,11.448)	
Lymphocyte	≤1.5×10^9^/L	(1.05, 0.39)			0.110
	>1.5×10^9^/L	(2.10, 0.91)	0.712	(0.469,1.080)	

IQR, interquartile range; HR, hazard ratio; CI, confidence interval; ECOG PS, Eastern Cooperative Oncology Group performance status; GCB, germinal center B cell; EBV, Epstein-Barr virus; CMT, chemotherapy; RT, radiotherapy; MSKCC, Memorial Sloan Kettering Cancer Center; LDH, lactate dehydrogenase; NLR, neutrophil-to-lymphocyte ratio; dNLR, derived neutrophil-to lymphocyte ratio; LLR, lactate dehydrogenase-to-lymphocyte ratio.

**Table 4:** Multivariate analysis of variables associated with overall survival.

Parameter		HR	(95%CI)	*P value*
LLR	≤166.8			***0.015***
	>166.8	1.792	(1.121,2.866)	
MSKCC	Low	–		***0.004***
	Intermediate	1.133	(0.613,2.096)	
	High	2.937	(1.459,5.908)	

HR, hazard ratio; CI, confidence interval; LLR, lactate dehydrogenase-to-lymphocyte ratio; MSKCC, Memorial Sloan Kettering Cancer Center.

### Survival Outcomes

**Figure 3:**
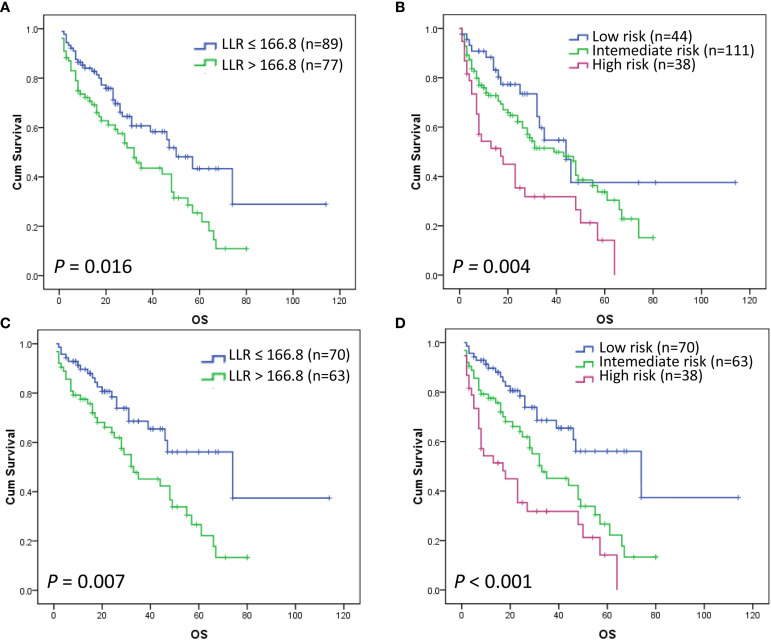
Kaplan–Meier curves for OS according to the LLR (*P* = 0.016) **(A)** and MSKCC score (*P* = 0.004) **(B)**. Overall survival Kaplan–Meier curves according to the LLR of low-intermediate MSKCC risk patients (*P* = 0.007) **(C)**. Kaplan–Meier curves according to the novel PCNSL LLR scoring system (*P* < 0.001) **(D)**.

Of all 248 patients, 193 patients had sufficient data to evaluate the prognostic significance of MSKCC, while 166 patients had both LLR data and MSKCC data. Most of the patients (n=103) died during the follow-up and the median OS was 33 months (95% CI 20.2–45.8). The 3-year and 5-year OS rates of the 193 patients were 48.4% and 30.2%, respectively. Patients with LLR ≤ 166.8 had a median OS of 50 months (n = 89), while those with LLR > 166.8 had a median OS of 32 months (n = 77) (*P* = 0.016) (**Figure 3A**). Although the OS rates of patients in the low-, intermediate-, and high-risk groups of the MSKCC scoring system were significantly different overall, with 5-year OS rates of 37.5% (n = 44), 33.7% (n = 111), and 14.1% (n = 38) (*P* = 0.004) (**Figure 3B**) (**Table 5**), there was no statistical difference between the low-risk and intermediate-risk groups (*P* = 0.184, data not shown). Similarity, the median OS of patients in the low-, intermediate-, and high-risk groups of the MSKCC scoring system were significantly different overall, with 5-year OS rates of 44, 39, and 17 months, respectively (*P* = 0.004) (**Table 5**), but there was no statistical difference between the low-risk and intermediate-risk groups (*P* = 0.184, data not shown).

**Table 5:** Survival outcomes of the assessable patients according to the different risk groups.

Models and risk groups	Patient, n	Median OS	5-Year OS rates	HR (95%CI)	*P value*
MSKCC score					*** 0.004***
Low-risk	44 (23%)	44	37.5%	–	
Intermediate-risk	111 (57%)	39	33.7%	1.461 (0.838,2.545)	
High-risk	38 (20%)	17	14.1%	2.687 (1.446,4.991)	
LLR					***0.016***
Low (≤166.8)	89 (54%)	50	43.4%	–	
High (>166.8)	77 (46%)	32	25.4%	1.710 (1.105,2.646)	
Low-intermediate MSKCC risk					***0.007***
LLR ≤ 166.8	70 (53%)	74	56.1%	–	
LLR > 166.8	63 (47%)	33	26.6%	2.082 (1.221,3.552)	

MSKCC, Memorial Sloan Kettering Cancer Center; OS, overall survival; LLR, lactate dehydrogenase-to-lymphocyte ratio; the median overall survival time is calculated in months.

### LLR Further Stratified Patients With Low and Intermediate MSKCC Risk

We further investigated whether LLR could stratify this latter group including patients with low and intermediate MSKCC scores. Our results showed that patients with LLR ≤ 166.8 had a significantly better OS than those with LLR > 166.8 within this group (*P* = 0.007) (**Figure 3C**) (**Table 5**). Based on this result, a novel three-tier PCNSL LLR prognostic scoring system was defined and evaluated. The criteria for defining the MSKCC high risk group were retained (i.e. age ≥50 and KPS <70), while the former low and intermediate risk patients defined according to the MSKCC criteria (i.e. age <50 or age ≥50 and KPS ≥70) were stratified by LLR. Accordingly, the resulting risk groups were so defined: low-risk (age <50 or KPS ≥70, and LLR ≤ 166.8), intermediate-risk (age <50 or KPS ≥70, and LLR > 166.8) and high-risk (age ≥50 and KPS <70). Outcomes of patients within the low-, intermediate-, and high-risk groups of the novel PCNSL LLR score resulted significantly different, with 5-year OS rates of 56.1% (n = 70), 26.6% (n = 63), and 14.1% (n = 38), respectively (*P* < 0.001) (**Figure 3D**) (**Table 6**). Similarity, the median OS of patients in the low-, intermediate-, and high-risk groups of the novel PCNSL LLR scoring system were significantly different overall, with 5-year OS rates of 74, 33, and 17 months, respectively (*P* < 0.001) (**Table 6**).

**Table 6:** The novel PCNSL LLR scoring system.

Model	Patient, n	Median OS	5-Year OS rates	HR (95%CI)	*P value*
PCNSL LLR score					***<0.001***
Low-risk	70 (41%)	74	56.1%	–	
Intermediate-risk	63 (37%)	33	26.6%	2.091 (1.226,3.565)	
High-risk	38 (22%)	17	14.1%	3.668 (2.072,6.493)	

OS, overall survival; the median overall survival time is calculated in months; low-risk (age <50 or KPS ≥70, and LLR ≤ 166.8), intermediate-risk (age <50 or KPS ≥70, and LLR > 166.8) and high-risk (age ≥50 and KPS <70).

### Validation of the Novel PCNSL LLR Scoring System

**Figure 4:**
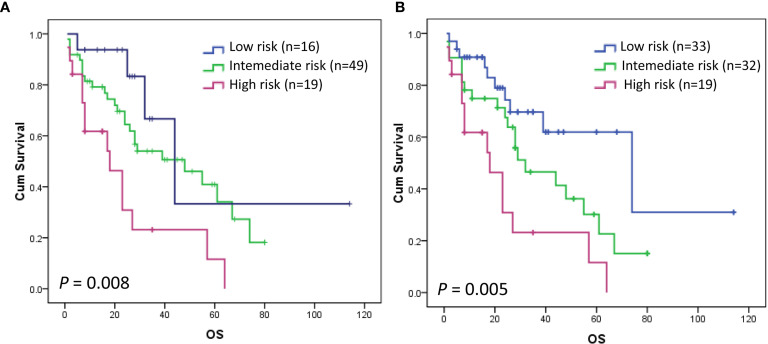
Kaplan–Meier curve for OS according to the MSKCC scoring system **(A)** and PCNSL LLR scoring system **(B)** in the validation cohort. The OS rates of patients in the low-, intermediate-, and high-risk groups of the MSKCC scoring system were significantly different overall, with 5-year OS rates of 33.3% (n = 16) and 40.9% (n = 49), and 11.6% (n = 19), respectively (*P* = 0.008). MSKCC scoring system could not discriminate patients within low-, and intermediate- groups in the validation cohort (*P* = 0.243) **(A)**. PCNSL LLR scoring system can clearly distinguish patients within the low-, intermediate-, and high-risk groups in the validation cohort (*P* = 0.005) **(B)**.

We randomly selected 124 PCNSL patients out of 248 for the validation purpose. Of the 124 patients, 84 patients had survival data and LLR data simultaneously. The prognostic score that we developed in the discovery stage was tested in the validation cohort. **Figure 4A** shows that the OS rates of patients in the low-, intermediate-, and high-risk groups of the MSKCC scoring system were significantly different overall, with 5-year OS rates of 33.3% (n = 16) and 40.9% (n = 49), and 11.6% (n = 19), respectively (*P* = 0.008). However, MSKCC score could not discriminate patients within low-, and intermediate- groups in the validation cohort (*P* = 0.243) (**Figure 4A**) (**Table 7**). The newly established score can clearly distinguish patients within the low-, intermediate-, and high-risk groups in the validation cohort, with 5-year OS rates of 61.9% (n = 33), 30.2% (n = 32), and 11.6% (n = 19), respectively (*P* = 0.005) (**Figure 4B**) (**Table 7**).

**Table 7:** Validation of the novel PCNSL LLR scoring system.

Model	Patient, n	Median OS	5-Year OS rates	HR (95%CI)	*P value*
MSKCC score					***0.008***
Low-risk	16 (19%)	44	33.3%	–	
Intermediate-risk	49 (58%)	48	40.9%	1.913 (0.665,5.503)	
High-risk	19 (23%)	18	11.6%	4.521 (1.476,13.848)	
PCNSL LLR score					***0.005***
Low-risk	33 (39%)	74	61.9%	–	
Intermediate-risk	32 (38%)	32	30.2%	1.920 (0.898,4.108)	
High-risk	19 (23%)	18	11.6%	3.899 (1.712,8.882)	

MSKCC, Memorial Sloan Kettering Cancer Center; OS, overall survival; the median overall survival time is calculated in months; HR, hazard ratio; CI, confidence interval; low-risk (age <50 or KPS ≥70, and LLR ≤ 166.8), intermediate-risk (age <50 or KPS ≥70, and LLR > 166.8) and high-risk (age ≥50 and KPS <70).

## Discussion

In our present study, univariate analysis showed that age, ECOG PS, treatment, MSKCC score, NLR, dNLR, and LLR were significantly associated with the OS of PCNSL patients. However, only MSKCC score and LLR were confirmed as predictors of OS by multivariate analysis. When LLR was introduced, patients within the low and intermediate risk groups, defined according to the MSKCC scoring system, were clearly separated into two groups with significantly different OS. Thus, the novel PCNSL LLR scoring system showed powerful prognostic value in PCNSL patients. Specifically, the performed ROC analysis allowed to determine an optimal LLR cut-off value of 166.8 as a relatively sensitive and specific prognostic marker for PCNSL.

Tumor cells prefer glycolysis to oxidative phosphorylation, even in presence of oxygen. This phenomenon, known as the Warburg’s effect, seems to be crucial for tumor development. Lactate dehydrogenase A (LDH-A) is a key enzyme in this process, which catalyzes pyruvate to produce lactic acid (20). In many cancers, the level of serum lactate dehydrogenase is an indirect marker of tumor hypoxia, neo-vascularization, metastasis development and poor prognosis (21). In addition, high expression of LDH is significantly related to the lack of lymphocyte infiltration at the invasive tumor border, to impaired host immune response, and to enhanced angiogenesis, resulting in poorer patient prognosis (22). Indeed, there is evidence that direct targeting of LDH is a potential therapeutic approach in cancer (23). Elevated serum LDH has been recognized as a poor prognostic indicator for hematological malignancies and solid tumors as well; moreover LDH has the highest prognostic impact among the International Prognostic Index (IPI) risk factors (24). Serum LDH is considered as an excellent surrogate marker of tumor burden in DLBCL patients. In addition, it is inexpensive and can be easily assessed during the routine clinical practice (14).

Host immune system, systemic inflammation and tumor microenvironment all contribute to malignancy development and progression (25). Recently, a low absolute lymphocyte count (ALC) has been proposed as a novel, independent prognostic parameter of poor survival in patients with NHL, such as DLBCL, ENKTL and follicular lymphoma (FL). One study specifically assessed ALC at diagnosis as a prognostic marker in PCNSL: Ji et al. analyzed 81 PCNSL patients who received high-dose methotrexate-based therapy and developed a new prognostic model including age >50 years, ECOG PS >1 and presence of lymphopenia. They successfully classified patients into low-, intermediate-, and high-risk groups observing 5-year OS rates of 74.3%, 21.7% and 12.5%, respectively (11). Decreased peripheral ALC may represent a surrogate biomarker of compromised anti-tumor host immunity in PCNSL patients. Lymphopenia may contribute to insufficient production of chemokines with anti-neoplastic activity such as interferon-γ, tumor necrosis factor, or interleukin-1. In addition, perivascular lymphocyte infiltration may be impaired in patients with lymphopenia and it can also increase the risk of treatment-associated complications which may affect patient survival (11). Based on these previous studies, we hypothesized that the ratio between a tumor burden marker (LDH) and an inflammation marker (lymphocyte count) (i.e. LLR), may represent an ideal prediction tool for estimating the outcome of PCNSL patients. Using a LLR cut-off of 166.8, our survival analysis stratified patients into two groups with median OS of 50 months (LLR ≤ 166.8) and 32 months (LLR > 166.8), respectively. To assess the importance of the specific LLR cut-off value, we also re-evaluated in our series a threshold which had been previously suggested for Extranodal NK/T Cell Lymphoma, Nasal Type (16), but we did not detect a survival difference between patients with low (≤128.44) and high (>128.44) LLR values (*P* = 0.143, data not shown). This observation highlights the importance of identifying and testing specific cut-off values for each tumor type.

To date, age and ECOG PS are the only two universally accepted prognostic parameters for PCNSL, indeed age >60 years and performance status >1 resulted clearly associated with a worse OS (26). A nationwide survey, which is the largest-scale PCNSL study to date, conducted on 466 patients treated at 62 Japanese institutions confirmed the above results (27). Recent studies have suggested that NLR is an independent prognostic parameter in patients with lymphomas, such as FL and DLBCL. In a study focused on NLR in PCNSL, Jung et al. demonstrated that NLR might play a potentially prognostic role in PCNSL by analyzing a cohort of 62 histologically confirmed cases. Nevertheless, no significant association was observed by multivariate analysis (28). Our study also identified a potential prognostic value of NLR in PCNSL, although the result was then not confirmed by multivariate analysis. Elevated dNLR level is an independent poor prognostic parameter in patients with multiple myeloma who are not suitable for transplantation (29). In DLBCL, an independent and significant correlation between dNLR and poor OS and DFS was reported (13). By univariate analysis, we found that high dNLR was associated with poor OS, but the real significance of this parameter has to be ascertained since it was also not confirmed by multivariate analysis. Nevertheless, this is the first time that the prognostic significance of pre-treatment dNLR is suggested in PCNSL.

Further efforts should be made to improve the prognostic efficacy of these scores, also tailoring them to patients treated with the current HD-MTX-based regimens (30). IPI, an internationally recognized prognostic scoring system for non-Hodgkin’s lymphomas, is commonly used to evaluate the prognosis of DLBCL and could represent a possibly alternative. However, the definition of number/sites of involvement and stage is controversial for PCNSL and thus it is rarely used in this setting. The MSKCC and the IELSG scores represent the most widely used prognostic models for PCNSL. In fact, the MSKCC score is a simplified version of the IELSG score, which takes into consideration more parameters beyond age and KPS. Unfortunately, many of these variables were not uniformly collected or reported in routine clinical practice, especially in older time periods. In addition, CSF examination is often avoided in PCNSL patients because of the potential risk of complications due to the increased intracranial pressure, thus CSF protein levels are not always available (31). For instance, one research enrolled 79 patients with intracranial PCNSL and successfully validated the MSKCC score, but failed to confirm the prognostic efficacy of the IELSG score, and in 9 out of the 79 patients (11%) the IELSG score could not be calculated due to the lack of CSF protein levels (31). Consequently, many PCNSL patients cannot be classified using the IELSG score during the daily practice while using the MSKCC score is always possible.

Some researchers also propose the Nottingham and Barcelona scoring system (NB), which is a three-factor scoring system, consisting of age, ECOG PS, presence of multifocal lesions or meningeal disease. It was developed in a relatively small cohort with 77 PCNSL patients that received CHOD/BVAM or BVAM chemotherapy regimens, so its value and application may be limited (32). Moreover, a study evaluated the validity of the existing scores: the IELSG, the NB, and the MSKCC in 182 newly diagnosed PCNSL patients. The IELSG and NB models showed poor separation for both PFS and OS, while the MSKCC score demonstrated a significant discrimination in terms of PFS in the training cohort. Based on these data, the IELSG, NB, and MSKCC scores showed an insufficient prognostic capability, although the MSKCC score performed better than the IELSG and NB models (33). These limitations of the proposed prognostic scoring systems highlight the importance of real-life feasibility when proposing novel prognostic models.

Based on the reasons above, our study only considered the MSKCC score as a control prognostic model. As expected, survival curves of the low-, intermediate-, and high-risk MSKCC groups varied significantly in our cohort, but no difference was observed between the low-risk and intermediate-risk groups, a finding which could be explained by the substantial changes in terms of therapeutic regimens which occurred since the development of the MSKCC score (11). Compared with the MSKCC score, LLR showed a superior prognostic value and could successfully classify patients into different risk groups: the novel PCNSL LLR scoring system was able to define three prognostically relevant groups of patients and in particular to further stratify the low/intermediate MSKCC risk groups. Therefore, the PCNSL LLR scoring system could represent a novel, objective, commonly available, cost-effective and accurate prognostic predictor of PCNSL outcome.

To our knowledge, the majority of the prognostic parameters for PCNSL have been validated by single center studies, which are inevitably subjected to an internal selection bias. Hopefully, the multicenter nature of our study helped control these biases, although it hampered the collection of fully comprehensive clinical data and thus it was not possible to analyze PFS, which is commonly used to prove the prognostic significance of a novel marker. Moreover, since the included patients were diagnosed within an extended period of time (2004-2019), the performed examinations and assessments varied in the different centers and time periods; in particular, some tests were not routinely done in the early years, and thus the IELSG score could not calculated. Finally, considering the low incidence of PCNSL, one major limitation of the present study is the lack of validation in the external cohort. This pitfall could be partially balanced by its multicenter nature and internal validation cohort which could support the general validity of the observed results, but this prognostic model should be validated in an independent external cohort and a multicenter prospective study is thus warranted in the near future to consolidate our findings.

## Conclusion

In the present study, we demonstrated the prognostic role of LLR in PCNSL patients. Besides, we proposed a novel PCNSL LLR-based scoring system which was able to improve the prognostic capability of the MSKCC model. The PCNSL LLR scoring system can be easily and routinely determined without additional expensive work-ups, thus it should be prospectively validated to enable its use in the daily practice, possibly also to tailor the therapeutic regimens of PCNSL patients.

## Data Availability Statement

The data analyzed in this study was obtained from the international PCNSL research collaboration consortium, the following licenses/restrictions apply: protection of patients' privacy according to ethical regulations. Requests to access these datasets should be directed to Dr. HY, E-mail: youhua307@163.com.

## Ethics Statement

The studies involving human participants were reviewed and approved by Ethics Committee of Cancer Hospital Affiliated to Guangzhou Medical University. The ethics committee waived the requirement of written informed consent for participation.

## Author Contributions

HY designed the study and contributed to manuscript revision. YG and LWe performed the research, analyzed and interpreted the data, and wrote the first draft of the manuscript. LT and LB contributed to manuscript preparation and revision. SK, LWa, YH, YZ, AP, PC, PT, AD, HZ, JL, and YW collected the data and contributed to manuscript preparation. All authors contributed to the article and approved the submitted version.

## Funding

The research activities are supported by the National Natural Science Foundation of China (81911530169 and 81903088). This work is also supported by the Innovation Support Program for Chongqing Overseas Returnees under Grant cx2019051, Chongqing Science and Technology Bureau under Grant/Award cstc2017jxjl130005.

## Conflict of Interest

The authors declare that the research was conducted in the absence of any commercial or financial relationships that could be construed as a potential conflict of interest.

## Publisher’s Note

All claims expressed in this article are solely those of the authors and do not necessarily represent those of their affiliated organizations, or those of the publisher, the editors and the reviewers. Any product that may be evaluated in this article, or claim that may be made by its manufacturer, is not guaranteed or endorsed by the publisher.
